# The Cause of Dr. Simpson’s Death

**Published:** 1871-01

**Authors:** 


The Cause of Dr. Simpson's Death.—Long a martyr to rheumatism, Sir James was, about two months ago, laid aside from active duty by a severe attack of *angina pectoris*, which recurred at uncertain intervals, and was accompanied by dyspnea, and latterly by some degree of dropsy. Though great danger was apprehended from the first, the issue was long and doubtful, and, up to a few days before his death, it was hoped that his valuable life might still be spared for some time, though a restoration to perfect health could not be expected. The end was, however, nearer than was supposed; and, after a few days of unconsciousness, he quietly breathed his last at ten minutes to eight, on the evening of Friday, the 6th of May. At the necropsy, the source of his sufferings and the cause of his death was found to be a large, dilated, fatty heart, globular in shape, and weighing eighteen ounces. At the apex of the left ventricle, the wall of which was thinned, an aneurism about the size of a pigeon's egg was discovered; all the other organs of the body were fatty. The arteries of the brain were atheromatous in a high degree. The brain itself, that imperial source of all his restless mental activity, was found to be by no means large; it weighed only fifty-four ounces, and was consequently but little above the average of forty-nine and a-half ounces. It may be remembered that the brain of Cuvier weighed above sixty-four ounces, and that of Abercrombie sixty-three, so that Simpson's brain forms rather an exception to the rule, that mental power depends upon size of brain. On the other hand it formed a remarkable example of the more incontrovertible fact, that mental vigor depends upon the number of convolutions and the quantity of grey matter; for, on being exposed, the brain presented an appearance not soon to be forgotten by those who were privileged to see it, in the apparently increased number of the convolutions and their great size and devolopment.—*Edin burgh Medical Journal.*

